# Identification of candidate genes associated with triple negative breast cancer

**DOI:** 10.18632/genesandcancer.147

**Published:** 2017-07

**Authors:** Audrey Player, Nissi Abraham, Kayla Burrell, Iria Ondo Bengone, Anthony Harris, Lisa Nunez, Telisa Willaims, Sharon Kwende, Wiley Walls

**Affiliations:** ^1^ Department of Biological Sciences, Texas Southern University, Houston, Texas, USA

**Keywords:** IL32, MYBL1, ETS1, GATA3, TMEM158

## Abstract

When triple negative breast cancer (TNBC) are analyzed by gene expression profiling different subclasses are identified, at least one characterized by genes related to immune signaling mechanisms supporting the role of these genes in the cancers. In an earlier study we observed differences in TNBC cell lines with respect to their expression of the cytokine IL32. Our analyses showed that certain cell lines expressed higher levels of the cytokine compared to others. Because TNBC are heterogeneous and immune-related genes appear to play a pivotal role in these cancers, we chose to examine the transcriptomes of the different cell lines based on IL32 expression. We performed group analyses of TNBC cell lines demonstrating high IL32 compared to low IL32 levels and identified IL32, GATA3, MYBL1, ETS1, PTX3 and TMEM158 as differentially associated with a subpopulation of TNBC. The six candidate genes were validated experimental and in different patient datasets. The genes distinguished a subset of TNBC from other TNBC, and TNBC from normal, luminal A, luminal B, and HER2 patient samples. The current project serves as a preliminary study in which we outline the discovery and validation of our list of six candidate genes.

## INTRODUCTION

Over the past few years significant amounts of research has been performed in efforts to characterize triple negative breast cancers (TNBC). The goal of these studies was to identify genes that would serve as biomarkers and/or candidates for targeted therapies. Even with all the efforts, there is still much to learn regarding the cancers. Data however are clear in demonstrating that the TNBC subtype represent a heterogeneous group of patients [1, 2], not just a single subtype. Lehman et al [3] identified 2188 genes that defined six TNBC subclasses that were designated as basal-like 1 (BL1), basal-like 2 (BL2), immunomodulatory (IM), mesenchymal (M), mesenchymal stem-like (MSL), and luminal androgen receptor (LAR) subclasses. Related to these studies Ring et al [4] developed a ‘leaner algorithm’ based on the same data and identified 101 genes that were able to identify the TNBC subclasses and in addition, predict patient outcome, recapitulating and expanding upon the results observed within the larger set of candidate genes. Together these data (a) emphasize the heterogeneity of TNBC (b) show that smaller gene sets can define the breast cancer subclasses and (c) demonstrate the ability of the gene sets to predict patient outcome.

The identification of an IM-like subclass in TNBC further validate the relationship between cancers and immune-related processes. Although this is a relatively recent observation in TNBC, its long been shown that inflammatory processes are associated with various types of cancers. One of the earliest observations came from Rudolf Virchow dating back to the 19th century when he observed the presence of ‘irritations’, now known to be inflammatory cells present in tumors. TNBC are particularly relevant to this discussion because these cancers often exhibit increased expression and changes in regulation of immune-related genes compared to estrogen receptor positive breast cancers [5, 6]. Data show significant levels of tumor associated macrophages (TAM) and tumor infiltrating lymphocytes (TIL) in TNBC compared to levels found in receptor positive patient samples. TAMs are thought to help in the proliferation of tumors [7, 8] and TIL [9] are thought to be the result of pro-inflammatory events. As confirmation of the relationship between receptor status and inflammation, studies show that Interleukin 1a (IL-1α), IL-1β, and IL-1ra [10] and IL6 [11] cytokines are over-expressed in TNBC patients compared to luminal cancers. In a separate study, Goldberg et al [12] found that elevated levels of IL6 in the serum and tissue were associated with invasiveness and poor prognosis. Denardo et al [13] reported that basal-TNBC with CD68-high/CD8-low levels correlate with overall poor survival. While these and other studies are instrumental in helping to understand the relationships between the immune system and TNBC, perhaps the most promising experiments of late involve studies of the programmed death 1 (PD1) and programmed death ligand 1(PDL1) membrane proteins associated with T-cells and cancer cells, respectively. PD1 and PDL1 (so called checkpoint inhibitor) genes are over expressed in melanoma, lung, stomach, head and neck and ovarian cancers [14-20] and treatment strategies that down regulate expression of these genes show an impressive response to therapies in clinical trials. Because TNBC demonstrate over-expression of immune-related genes compared to other breast cancer subtypes, the cancers are being examined for the possible utility of PD1/PDL1 directed therapies. Mittendorf et al [21] found that PD1 was expressed in approximately 20% of TNBC, making the gene a possible therapeutic target in the cancers. Recent studies by Beckers et al [22] however show that PDL1 in TNBC is associated with improved clinical outcome. While these and other data convincingly support the relationship between immune related processes and TNBC, they underscore the need to better understand the signaling mechanisms involving immune-related processes and these cancers.

In an earlier study, we identified Interleukin 32 (IL32) as differentially expressed in TNBC compared to non-tumor and luminal breast cancer cell lines and patient samples [23]. Data however showed that only certain TNBC displayed IL32 gene over-expression. Because TNBC are known to be heterogeneous, the goal of this study is to start by characterizing TNBC with respect to IL32 expression and based on bioinformatics-based assessment, identify genes that are differentially expressed in TNBC cell lines that are high in IL32 compared to low in IL32 gene expression. Once these genes were identified, as a final selection criteria genes were selected based on experimental validation and assessment of differential expression in non-tumor, luminal and HER2 breast samples. Applying this selection criteria, a short list of 21 genes were identified and a final list of six genes were selected. The cell lines and patient samples analyzed in this study were available as publically available DNA microarray datasets and the initial gene discoveries were performed using unsupervised methods of analyses. Our gene candidates were validated following PCR analyses and by supervised analyses of the Maire [24] and other datasets. The Maire dataset contained gene expression information from normal, luminal A, luminal B, HER2 and TNBC patient samples. A final list of six candidate genes were found to be differentially expressed in a subpopulation of the TNBC compared to all other TNBC, normal, luminal A, luminal B and HER2 patient samples. The genes included IL32, PTX3, GATA3, TMEM158, ETS1 and MYBL1. The current study details our approach for identification and selection of our list of genes, followed by validation of their gene expression levels in cell lines and clinical samples.

## RESULTS

### Selection and analyses of microarray datasets demonstrating IL32-high vs IL32 low levels

The current study involved analyses of both patient samples and cell lines (Table 1). An outline of our experimental approach is given in Table 2. The study began with the observation that only particular basal-like\TNBC were positive for IL32 gene expression while other cell lines showed lower to negligible levels. Our aim was to separate the basal-like\TNBC cell lines into two groups based on IL32 expression and perform differential expression analyses and identify genes associated with the two groups. The basal-like\TNBCs were extracted from Gse12777 and Gse34211 datasets, each containing 18 and 24 cell lines respectively. To better assess the reproducibility of IL32 expression in the various samples, only cell lines common to both datasets were considered for analyses. Nine cell lines were found to be common in both datasets and in all, IL32 demonstrated a similar pattern of gene expression. Of the nine cell lines, six cell lines (i.e., HCC1569, HCC1143, MDA MB436, Cal851, Hs578T and MDA MB231 to a comparatively lesser degree) demonstrated higher IL32 levels, and three (HCC70, HC1806, and BT20 expressed lower to negligible IL32 levels [23]. Except for HCC1569 (which is positive for HER2), all other cell lines were designated as TNBC. The receptor status of the cell lines is well documented. The cell lines could not otherwise be stratified based on Basal A and Basal B subtype. Analysis of the same cell lines in Cancer Cell Line Encyclopedia (CCLE) [25] showed a similar pattern of IL32 expression (data not shown).

**Table 1:** Summary of the publically available GEO dataset used in the study

Gene expression omnibus	Type	Characteristics\subtype	Number of samples (n)	Publication
Accession number				
GDS2250\GSE7904	Patients	Normal\non-basal-like\basal	*n* = 47	Richardson AL, Wang ZC, De Nicolo A, et al Cancer Cell. 2006 Feb;9(2):121-32.
GDS1329	Patients	Apocrine\basal-like\Luminal	*n* = 49	Farmer P, Bonnefoi H, Becette V, et al Oncogene. 2005 Jul 7;24(29):4660-71.
GSE34211	Cell lines	Luminal\basal	*n* = 35	Hook KE, Garza SJ, Lira ME, et al Mol Cancer Ther. 2012 Mar;11(3):710-9.
GSE12777	Cell lines	Luminal\basal	*n* = 49	Hoeflich KP, O'Brien C, Boyd Z, Clin Cancer Res. 2009 Jul 15;15(14):4649-64.
GSE65194	Patients	Normal, Luminal A, Luminal B Her2, Triple negative	*n* = 161	Maire V, Némati F, Richardson M, Cancer Res. 2013 Jan 15;73(2):813-23.
GSE76124	Patients	Triple negative, normal	*n* = 48	Burstein MD, Tsimelzon A, Poage GM, et al Clin Cancer Res. 2015 Apr 1;21(7):1688-98.
GSE43502	Patients	Triple negative	*n* = 25	Yu KD, Zhu R, Zhan M, Rodriguez AA, et al Clin Cancer Res. 2013 May 15;19(10):2723-33

**Table 2:**
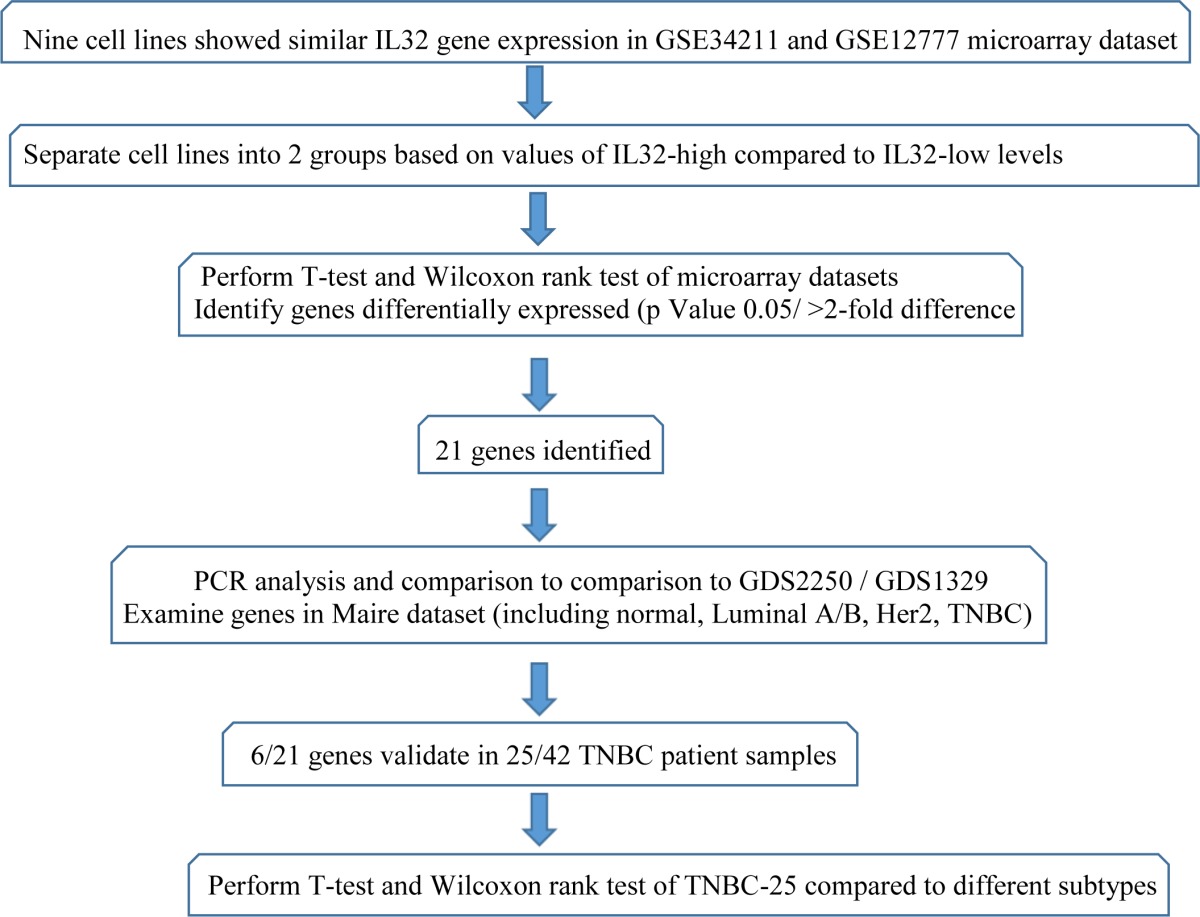
Outline of the experimental approach used for gene discovery and validation

We separated the Basel-like\TNBC cell lines into two groups defined by high compared to low IL32 gene expression levels, and performed T-test and Wilcoxon rank analyses to identify the genes that defined the two groups. Gene expression levels were filtered to select genes demonstrating at least a 2-fold difference with p values <0.05. A list of 21 genes were selected for study. These genes were designated as those differentially expressed in the IL32-high compared to the IL32-low basal/TNBC cell lines. The complete list of 21 genes and their pattern of differential gene expression in the cell lines are given in the supplemental Table 1. Search Tool for the Retrieval of Interacting Genes/Proteins (STRINGTM) analysis [26] show that the 21 genes are enriched in inflammatory and cytokine responses (p value 0.0059) and in the pathway related to HTLV infection (p value 0.0031) (Supplementary Figure 1). The association with immune-related processes was not surprising because the initial analysis were performed to compare cell lines based on IL32-high compared to IL32-low levels of the cytokine gene.

### Selection of the final list of six candidate genes based on PCR

**Figure 1:**
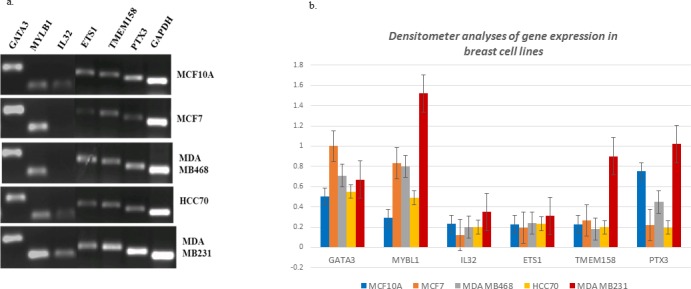
PCR validation of the 6 candidate genes (a) PCR (b) densitometer analysis agarose gel profile.

In efforts to validate the genes and identify a reliable subset of candidate genes, the list of 21 genes were examined experimentally via PCR using MCF7 (luminal), MCF10A (triple negative, non-tumor), MDA MB468 (TNBC, basal A), (HCC70 (TNBC, lower IL32) and MDA MB231 (TNBC, higher IL32) cell lines. The focus was to validate differential expression of our candidate genes in IL32 high compared to IL32 low cell lines, but at the same time determine the gene expression levels in certain other breast samples. HCC70 was chosen to represent the IL32-low cell line and MDA MB231 was chosen to represent the IL32-high cell line based on previous observations showing a differential pattern of expression between the cell lines in the original analyses. Following PCR analyses, and based on levels observed for HCC70 compared to MDA MB231, 6 of the 21 genes demonstrated differential gene expression (Figure 1), thus chosen as candidate genes for downstream analyses. The final list of genes included IL32 (cytokine), PTX3 (tumor necrosis factor induced gene), GATA3 (transcription factor), TMEM158 (transmembrane protein), ETS1 (transcription factor) and MYBL1 (transcription factor). All of the genes were up-regulated in IL32-high TNBC (MDA MB231) except GATA3 where comparably lower levels were detected. These data are consistent with that documented by other investigators demonstrating lower levels of GATA3 in some TNBC (i.e., MDA MB231), and significantly higher levels of GATA3 in luminal samples (i.e. MCF7) [27]. Significant levels of PTX3 gene was observed in MCF10A, but still higher levels were found in MDA MB231 compared to all other cell lines. A summary table of the Gene Ontology (GO) as generated by Gene Annotation Tool to Help Explain Relationships (GATHER) [28] and mAdb [29] are given in Table 3. GATHER and mAdb analyses show our gene panel over-represented in response to defense mechanisms, immune responses and protein binding/transcription factor activity. GATHER show highly significant p values and mAdb show a higher than expected functional occurrence for our gene set, validating enrichment of the cellular processes. These data also suggest ETS1, IL32 and PTX3 are regulated by NFKappa B (p65), and validate protein binding of both GATA3 and ETS1.

**Table 3:** Gene Ontology functional analysis of the 6 candidate genes as designated following GATHER and mAdb program analyses

**GATHER**			
GENE ONTOLOGY			
Annotation	genes	significance	bayes factor
defense mechanism	IL32, PTX3, GATA3, ETS1	0.0001	7
immune responses	ETS1, IL32, PTX2	0.0003	4
kegg pathway	none		
transcription factor NFKAPPA B (p65)	IL32, ETS1, PTX3	0.0001	5
protein binding ETS2	ETS1, GATA3	<0.0001	11
**mAdb**			
GENE ONTOLOGY			
Annotation		Observed/Expected Ratio	
immune responses	IL32, PTX3, GATA3, ETS1	6.9	
transcriptional activity	ETS1, GATA3, MYBL1	9.6	

### Analyses of the six candidate genes in Maire DNA microarray dataset

As validation of the processes used to select our candidate genes, the expression pattern of the 6 genes were analyzed against the Maire clinical dataset [24], which contained normal, luminal A, luminal B, HER2 and TNBC patient samples. This dataset was chosen based on its sample types and the platform used for the microarray analyses. A description of the Maire dataset can be retrieved from GEO GSE65194. Seven normal samples from the GDS2250 (GSE10780) dataset were combined with the Maire dataset in order to increase the number of normal patient samples. The quality metrics (i.e., % present, 3/5’ ratio, and scaling factor) for both GDS2250 and Maire microarray datasets were similar, allowing for the datasets to be combined. The datasets did not show evidence of a batch effect when they were compared via cluster analysis. In total, 168 unique clinical samples were used for the analyses including 37 normal, 29 luminal A, 30 luminal B, 30 Her2 and 42 TNBC from the Maire and GDS2250 datasets. The clinical samples in both datasets were defined previously by diagnoses and immunohistochemistry processes.

**Figure 2:**
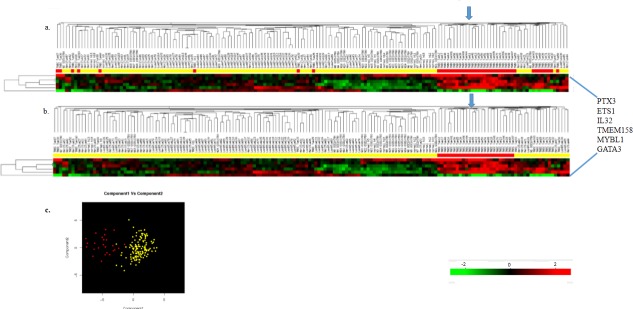
Hierarchical cluster (HC) analysis of 6 candidate genes in Maire patient samples (a) HC analyses of 6 genes in all TNBC (red bars) compared to normal, Luminal A, B and HER2 (yellow bars). (b) HC analyses of 6 genes in TNBC-25 compared to normal, Luminal A, B and HER2 patient samples. The arrow shows clustering of the TNBC-25 patient samples. (c) Principal component analysis (PCR) of HC identified in panel B – TNBC-25 compared to normal, Luminal A, B and HER2.

T-test were performed to compare the TNBCs (i.e., group 1) compared to all the other clinical sample types (i.e., group 2) using an unsupervised method of analysis. Results of the analysis were then used to access the expression of our 6 candidate genes (Figure 2a). These data show the six genes appear to define 25 of the 42 TNBC but not all TNBC. When the samples are regrouped such that the 25 TNBC (TNBC-25) represent a separate group-1 and all other TNBC, luminal A, luminal B, HER2 and normal to represent group-2, data show the six genes differentially associated with TNBC-25 (Figure 2b). Results of these analyses show that our 6 candidate genes are differentially associated with ~60% of the TNBC. Data by other investigators show that TNBC are a heterogeneous subtype [2]. Based on our studies, we suggest that our 6 candidate genes show differential expression in some TNBC, in part supporting the differences related to the TNBC genomes.

Although PTX3 was a somewhat reliable candidate for characterizing TNBC, substantial levels of the gene were identified in some normal samples, causing the normal patient samples to cluster closely to TNBC. Significant levels of PTX3 were detected in several of the normal samples, but the levels did not exceed those observed for TNBC. When PTX3 was excluded from the gene list and the cluster analyses repeated, normal samples clustered at opposite ends, most distant from the TNBC (data not shown). We should note that while ETS1 failed to demonstrate at least a 2-fold difference when examined against our entire patient sample population, it consistently demonstrated highly significant p values, and did appear differentially expressed upon analysis by PCR.

The expression of our candidate genes in the TNBC-25 were also compared directly to the levels found in normal, luminal A, luminal B and HER2 (Table 4). Even with PTX3 included in the analysis, statistically significant differences were observed between the TNBC-25 and normal samples, and luminal A, B and HER2 samples.

**Table 4:** Differential gene expression of 6 genes in TNBC-25 compared to all Luminal A/B, Her2, normal samples

Affymetrix probe-ID	Gene symbol	Fold difference	p value
	up-regulated TNBC-25 vs all samples		
206157_at	PTX3	2.64	2.20E-08
213906_at	MYBL1	2.88	2.70E-08
213338_at	TMEM158	2.43	6.29E-11
203828_s_at	IL32	2.00	2.10E-09
1555355_a_at	ETS1	1.43	1.80E-08
	down-regulated TNBC-25 vs all samples		
209604_s_at	GATA3	2.40	1.25E-09

We chose to also examine gene expression levels of our candidate genes in the GDS2250 and GDS1329 patient sample datasets. The datasets were chosen based on their use of the U133 plus 2 platform and because the gene expression profiles for the Affymetrix probes-set were curated and available in Gene Expression Omnibus [30]. GDS2250 contained 7 normal (referenced above), 19 luminal and 20 basal-like/TNBC samples. The GDS1329 contained 16 basal-like/TNBC, 27 luminal and 6 apocrine tumors. Neither of the datasets specifically distinguished basal-like compared to TNBC samples, nonetheless, our candidate genes showed a specific pattern of over-expression expression in some basal/TNBC compared to luminal patient samples (Supplementary Figure 2).

### Analyses of the candidate genes in additional datasets

In efforts to further validate our candidate genes, we compared our gene list to two additional TNBC datasets. The first dataset was the result of genomic profile studies aimed at defining the heterogeneity of TNBC. Burstein et al [31] found that TNBC gene expression patterns can be defined by four subgroups which they designated Luminal androgen receptor (AR; LAR; Subtype 1) Mesenchymal (MES; Subtype 2) Basal-Like Immune-Suppressed (BLIS; Subtype 3) Basal-Like Immune-Activated (BLIA; Subtype 4). LAR are characterized based on estrogen receptor and receptor regulated genes (ESR1, GATA3). MES are characterized by mesenchymal-stem like and claudin low associated genes. BLIS are characterized by genes involved in down-regulation of B-cell, T-cell, and natural killer cell immune-regulating pathways and cytokine pathways, and in patients with the worst disease free survival. BLIA are characterized by tumors displaying an up-regulation of genes controlling B-cell, T-cell, and natural killer cell functions and in patients with an overall better prognosis.

**Figure 3:**
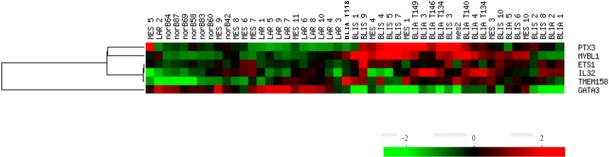
Analyses of 6 candidate genes in normal, and TNBC characterized as MES, LAR, BLIA and BLIS

As itemized in GEO, we chose the first samples from each group for download, including 7 normal patient samples, 10 LAR, 11MES, 10 BLIS and 11BLIA samples identified as belonging to the particular subgroup. Analyzing our candidate genes in these samples, we observed a distinct clustering pattern however, the most distinct clustering pattern was observed for both BLIA and BLIS compared to MES and LAR (Figure 3). Both the BLIA and BLIS subgroups are characterized by an over-representation of genes involved in immune processes, similar to the GO designations of our gene panel. Even though our genes fail to serve as signatures for a particular TNBC subclass, the results are somewhat consistent with our previous observations, in that our genes likely recognizes TNBC over-represented by genes related to immune processes. Our 6 candidate genes were not identified in the Burstein et al study.

As validation of the TNBC samples, we analyzed the Maire TNBC using the 101-gene list previously shown by Kao et al [32] to be TNBC gene expression signatures. All but four of the Maire samples showed precise correlation (via clustering) with the Kao 101-gene list (data not shown). The Kao list of signatures were selected because their data were generated following analyses of both cell lines and clinical samples, similar to the approach outlined here. Their approach and others [33] validate the use of use of cell lines as a tool for gene discovery.

We also compared our gene list to a small TNBC dataset defined by patient samples that were resistance to neoadjuvant chemotherapy [34]. The investigators theorized that after neoadjuvant chemotherapy, chemoresistant TNBC patients that relapse (i.e., recurrent) compared to those that do not relapse (i.e., no recurrence) represent different populations of TNBCs distinguishable by gene expression profiling. We grouped the patient populations into two groups based on their designation as recurrent (R) compared to non-recurrent (NR) and performed supervised analysis of our six candidate genes. Our gene list failed to distinguish the TNBC patient populations (data not shown). As a result of their analyses, the investigators identified a list of 7 genes that distinguished the R compared to the NR TNBC populations, one of which included GATA3 which was a gene common to both their and our candidate-lists.

### Comparison of the TNBC-25 to other TNBC samples in the Maire dataset

**Figure 4:**
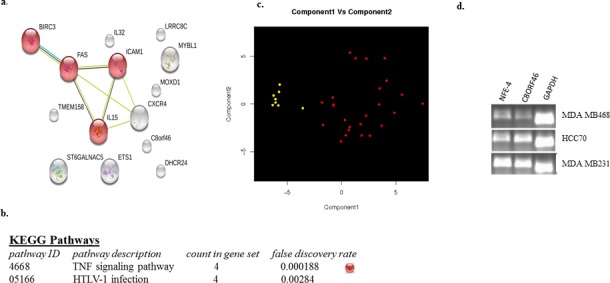
Genes identified when TNBC-25 compared to the 8 other TNBC (a) STRING analysis of 16 genes differentially expressed between the two groups of TNBC. (b) KEGG analysis of panel of 16 genes. Red ball shows genes involved in TNF pathway in panel a. (c) PCA analysis of the panel of 16 genes. (d) gel electrophoresis of NFE4, C8orf46 and GAPDH (control) transcripts in MDA MB468, HCC70 and MDA MB 231.

The TNBC-25 were selected based on cluster analyses of the total number of TNBC following analysis of our candidate genes (see Figure 1). Our data do not suggest that the TNBC-25 represent a distinct subpopulation of TNBC, however data do suggest that (a) a sizeable number of the TNBC can be distinguished based on our gene panel, and (b) the gene panel can distinguish TNBC compared to normal luminal A/B and HER2 clinical samples. However, a key question is ‘how do the TNBC-25 differ from the remaining TNBC’? To begin to address this question we compared the TNBC-25 to the remaining TNBC (including 17 TNBC) and observed minor differences between the two groups. This is because even though the TNBC-25 appeared as a cluster, there were several other TNBC clustering quite close which demonstrated a similar gene expression profile based on our genes. When we compared the TNBC-25 to the TNBC which clustered furthest from the TNBC (which included 8 TNBC; Figure 1), more substantial differences were observed between the transcriptomes (Table 5). The final gene list contained 16 genes when a less stringent cut-off of 1.5 fold difference and a more stringent p value of 0.001 was used. The stringency related to fold-difference was lowered because we expected the two groups of TNBCs to be very similar. Despite the fact lower levels of differential gene expression were detected in many of the samples, the genes still appeared to define TNBC as determined by clustering and PCA analysis. Four of the 16 genes were from our original gene list which included MYBL1, IL32, TMEM158 and ETS1. GATA3 and PTX3 are not included in the list of 16 genes, perhaps because GATA3 is a luminal cell marker, and even though PTX3 show differential expression in TNBC, the gene shows substantial expression in some normal samples. STRINGTM analysis show enrichment of the gene list in Tumor necrosis factor (TNF) pathway with a false discovery rate of 0.0001 (Figure 4). There were four genes (ICAM1, IL15, BIRC3 and FAS) found to be associated with the TNF signaling pathway, but although the p values and false discovery rates were significant, we chose to on experimentally validate genes with > 2.0 fold differences. The genes linked by STRINGTM were found to be associated via known interactions based on curated databases, text mining and co-expression. The NFE4 and TRIL genes were not detected by the STRINGTM program, so they were excluded from these analyses. Together these data support the association of MYBL1, IL32, TMEM158 and ETS1 with certain TNBCs and the enrichment of genes involved in immune related processes as differentially associated with the cancers.

**Table 5:** TNBC-25 compared to select TNBC based on T-test analysis of microarray datasets

Affymetrix ID	Gene symbol	Fold difference	p value	Description
220979_s_at	ST6GALNAC5	2.6	1.40E+06	ST6 N-acetylgalactosaminide alpha-2,6-sialyltransferase 5
229430_at	C8ORF46	2.2	4.70E+05	chromosome 8 open reading frame 46
1560527_at	NFE4	2.5	8.50E+07	Transcription factor NF-E4
213338_at	TMEM158	2.1	8.20E-08	transmembrane protein 158 (gene/pseudogene)
216252_x_at	FAS	1.9	1.30E-04	Fas cell surface death receptor
203828_s_at	IL32	1.9	2.30E-04	interleukin 32
210538_s_at	BIRC3	1.8	2.00E+06	baculoviral IAP repeat containing 3
213906_at	MYBL1	1.8	2.30E-04	MYB proto-oncogene like 1
1555355_a_at	ETS1	1.6	1.99E+05	ETS proto-oncogene 1, transcription factor
1554474_a_at	MOXD1	1.6	4.00E-04	monooxygenase DBH like 1
205992_s_at	IL15	1.5	1.80E-03	interleukin 15
228314_at	LRRC8C	1.5	1.10E+06	leucine rich repeat containing 8 family member C
202638_s_at	ICAM1	1.5	1.20E-03	intercellular adhesion molecule 1
211919_s_at	CXCR4	1.5	1.90E-03	C-X-C motif chemokine receptor 4
205150_s_at	TRIL	−1.7	7.10E-03	TLR4 interactor with leucine rich repeats
200862_at	DHCR24	−2.1	5.30E+05	24-dehydrocholesterol reductase

In addition to MYBL1, IL32, TMEM158 and ETS1, twelve other genes were identified, however, only 4 of the 12 genes demonstrated significant differences (i.e., > 2-fold) between the two TNBC groups allowing for their analysis via PCR. The genes included STGALNAC5, NFE4, C8ORF46 and DHCR24. Using MDA MB231, HCC70 and MDA MB468 as test samples, only the NFE4 demonstrated a consistent differential expression pattern similar to that generated by data analyses. NFE4 was upregulated in MDA MB231 (basal B) compared to HCC70 and MDA MB468. Although both HCC70 and MDA MB468 are basal A samples (and MDA MB231 is basal B), preliminary data suggest that our gene panel does not distinguish based on basal A/basal B typing. These data suggest NFE4 might be a suitable gene to examine further as validation of its differential pattern of expression in TNBC.

## DISCUSSION

In an earlier study we found that the cytokine IL32 was over-expressed in particular TNBC and under-expressed in other TNBC cell lines. Based on this observation, the primary goal of the current study was to further characterize TNBCs based on their expression of IL32 and identify genes differentially expressed with IL32 with the aim of identifying possible biomarkers. Data by other investigators support the involvement of immune-related genes in both pro-inflammatory and anti-inflammatory processes in cancers [35], and these and other studies show that the heterogeneity in TNBCs can in part be due to these complex immune-related processes [36]. Our rationale was that characterization of TNBC samples based on IL32 expression might lead to identification of genes relevant to immune processes, and this might ultimately result in a better understanding of signaling processes that either drive or are associated with the disease. We were in search of genes that were reliably differentially expressed across various datasets, and genes that would validate via experimental analyses. Our main focus is to continue analyses of TNBC, and also identify genes that might distinguish TNBC from normal and other breast cancer types. We ultimately selected six genes for study including IL32, PTX3, GATA3, ETS1, TMEM158 and MYBL1. Also, following a separate analyses, IL32, ETS1, TMEM158 and MYBL1 showed differential gene expression when our subset of TNBC-25 were compared to other TNBC patient samples. For this particular subset of genes, the difference was not expected to be as substantial (i.e., > 2-fold) as when TNBC was compared to luminal A, luminal B, HER2 and normal patient samples, however the false discovery rate and p values for the expression patterns of these genes was quite significant. Regardless of the comparisons, when the TNBC-25 are compared to other groups, even when we increase stringency related-to-fold-difference of these comparisons, the gene lists continue to show over-representation of genes related to immune processes as determined by GO analyses. It is too early in our studies to suggest that the heterogeneity of TNBC can be defined by IL32 or even the other five genes described in this study. However, we can say that our panel of genes demonstrate a specific pattern of differential expression in certain TNBC, and in TNBC compared to normal and luminal breast samples. Three of the six genes are transcription factors and may play a role either synergistically or antagonistically as key regulators in TNBCs. In the event these genes continue to show promise, the genes will be examined individually and in combination to determine their role in TNBC.

The IL32 gene has nine isoforms several of which appear to contribute to the differential gene expression [37] observed in TNBC. To the best of our knowledge, Park et al [38] were the first to demonstrate a role of IL32 gene in breast cancer. Park et al detected IL32 β in both MDA MB231 and MCF7 cell lines, and showed that both samples were regulated by vascular-endothelial-growth-factor-signal-transducer-and-activator (VEGF-STAT3). Their data also showed that IL32 contributed to tumor progression in both cell lines.

In addition to studies by Park et al, over the past few years there has been a substantial increase in the number of published studies related to the function and role of IL32 in cancers [39-41]. Data show that IL32 is a pluripotent cytokine that induces IL6 and Tumor necrosis factor alpha [42]. We found that Tumor necrosis factor receptor super family genes (TNFRSF) and IL6 were highly expressed in our PCR analyses, along with IL8. IL6, TNFRSF and IL8 showed differential expression via PCR analysis, but all failed to show reliable differential patterns of expression when examined in patient samples. As a result, The TNFSF, IL6 and IL8 genes were eliminated from our candidate gene list. IL32 gene however appears to demonstrate differential expression when compared across different types of breast cancers.

Based on their level of differential expression across patient samples, data suggest that MYBL1 and TMEM158 are candidates worthy of further study. Kao et al [32] identified down-regulation of the transcriptional regulator MYB in TNBC, while in our studies we identified MYBL1 transcription factor as over-expressed in TNBC compared to most other normal and breast cancer types. MYBL1 belongs to MYB family and has been shown to function as an antagonist of MYB. While MYB and MYBL2 were both down-regulated in TNBC samples processed here, neither made our final gene list. Data suggest MYB is a tumor suppressor while it might be that MYBL1 is involved in tumor progression. Liu et al [43] validated the role of MYB as a ‘good prognostic indicator’ in breast cancer and using bioinformatics-based approaches suggested that MYBL1 could partially decrease the activity of MYB. In the event the MYBL1 gene continues to demonstrate differential expression in breast clinical samples following RNA and protein analyses, its precise mode of action will be studied for its association with a subpopulation of TNBC. As for TMEM158, the gene is a transmembrane protein also described as RAS-induced senescence protein 1. The gene has been shown to be associated with different types of cancers. Studies show that TMEM158 frame-shift mutations are frequently detected in colorectal cancer [44] and other studies show over expression of the gene in 15% of primary breast carcinomas [45]. Little is known about the function of the protein, but it could be that the breast cancers previously described as overexpressingTMEM158, represent an enrichment of TNBC.

A substantial difference in expression was observed when our TNBC-25 were compared to normal and luminal clinical samples, with marginal differences between the TNBC-25 and the remaining TNBC. Compared to other groups, slightly higher levels of IL32 gene were detected in HER2 breast cancers. Overall, TMEM158, Mybl1, IL32, ETS1 and PTX3 gene levels were higher in TNBC. Strategies focused on down-regulating TMEM158, Mybl1, IL32, ETS1 and PTX3 should be considered to determine their effect on TNBC tumor progression. These experiments should lead to a better understanding of signaling events that define TNBC. Although PTX3 shows statistically significant differential expression in our studies, it also shows expression in some normal breasts which make it a less desirable candidate compared to the other gene candidates. GATA3 gene, on the other hand, has been extensively studied in breast cancers. Our interest lie in the possibility that GATA3 gene is related to indirect cell signaling mechanisms with one or more of the five candidate genes.

A key question related to this study is “how or if the six candidate genes are related” in TNBC processes? Based on pathway analysis, the six genes did not show relationships based on signaling events, however, GO and STRINGTM analyses did show a higher than expected ratio of association with immune-related processes as determined by text-mining, experimental assessments and co-expression assays. In this current study we observe opposing levels of expression of GATA3 and ETS1, however, there is evidence that GATA3 and ETS1 function synergistically to regulate inflammatory processes [46]. And, in a separate study Jung et al [47] show that ETS1 is related to mechanisms that promote aggressiveness and poor prognosis in TNBC. Demonstrating a possible link to IL32, ETS1 has been shown to regulate VEGF which has been shown to regulate IL32 gene expression. Although it has not been substantiated, based on GATHER analyses performed in this study NFKappa B might play a role in regulating IL32 PTX3 and possibly ETS1. Demonstrating a possible link to TMEM158 (which is a RAS-induced gene), recent data show that ETS1 is required for activation of RAS/ERK signaling pathways [48]. Expansion of the STRINGTM network analyses to show ‘more (+)’ indirect associations, show experimental, text-mining and co-occurrence relationships between MYBL1, GATA3 and ETS1. Differential expression of these genes in our study, further support their role in mechanisms related to TNBC. As for how our candidate genes contribute to the biological functions in TNBC remains to be determined.

## MATERIALS AND METHODS

### Cell lines

The cell lines used in the study were MCF10A, MCF7, MDA MB468, HCC70 and MDA MB231. The cell lines were purchased from Atcc.org (Manassas VA). Except for MCF10A, the cells were grown in Dulbecco's Modified Eagle Minimum essential media (DMEM) supplemented with 1% penicillin and 10 % serum in a 370C incubator with 5% CO_2_ as suggested by the supplier. MCF10A cells were grown in DMEM / F12 (Gibco) supplemented with 10% FBS, 20ng/ml epidermal growth factor, 20ng/ml insulin-like growth factor and 500ng/ml hydrocortisone. Cells were fed twice weekly, grown to 70-90% confluence and harvested using a 0.25% trypsin solution.

### Microarray datasets for cell lines and clinical patient samples

The datasets used in this study were identified in Gene Expression Omnibus (GEO) [30] using the search terms “Affymetrix and breast and/or triple negative”. The datasets selected for study were generated using the Affymetrix U133 plus 2.0 microarray platform. This microarray platform would allow for analyses of the transcriptome and down-stream signaling pathway studies. A summary of the cell line and patient sample information is given in Table 1. The Affymetrix microarray quality metrics (i.e., box plots, 3/5’ ratios, scale factors, background and percent present genes) were examined to ensure the quality of the datasets.

### RNA extraction and cDNA generation

Total RNA was extracted from cell lines using the TRIzolTM reagent, followed by further purification using the QIAGEN RNA extraction kit; both methods were performed as suggested by their manufacturer. RNA integrity was assessed by spectrophotometer (i.e., A260/A280 ratios) and via RNA agarose gel analyses. The cDNA was generated using the iScript Reverse Transcriptase (BioRad.com; Hercules CA) kit as suggested by the manufacturer using a starting concentration of 1 microgram of total RNA.

### PCR primer design and polymerase chain reaction (PCR)

#### Primer design

The sequence corresponding to each gene (in the form of probe-set information) was retrieved from NetAffxTM resources (http://www.affymetrix.com/estore/analysis/index.affx) available at Affymetrix.com. The same information was available as part of the mAdb data analysis portal (https://madb.nci.nih.gov/) [29]. PCR primers were designed using the Primer3TM program (http://bioinfo.ut.ee/primer3-0.4.0/) [49], using the default primer design conditions. The primer sequences were validated using the University of California at Santa Cruz In Silico PCR program [50] and National Biotechnology Blast program (http://www.ncbi.nlm.nih.gov/) [51] primer programs. The primer sets corresponding to each gene were synthesized by IDTDNA (IDTDNA.com, Iowa). Primer sequences are summarized in Supplementary Table 2.

#### PCR reaction

Approximately 10 nanograms of cDNA was mixed with the PCR primer (~10uM) and TAQ polymerase Masters mix (Life Technologies) as suggested by the manufacturer. All PCRs were run at 95degrees (5min), and 30-32 cycles at 95degrees 30seconds, 58degrees 30seconds, 70degrees 30seconds. Qualitative PCR was performed to determine the relative difference in gene expression levels between the cell lines. The PCR products were analyzed using 1-2% agarose gels and the relative difference in transcript levels per cell line were determined by densitometer analysis of the agarose gel profiles using the ImageProTM software available on the Bio-Rad ChemiDocTM.

### Data analyses

#### Analysis of TNBC cell lines and breast cancer clinical samples

Datasets were retrieved from the GEO available on the National Center for Biotechnology Information (NCBI). Raw data included in the CEL intensity files for each dataset were uploaded into mAdb data analyses resource (https://madb.nci.nih.gov/) available through the National Cancer Institute. Resources available at mAdb include but not limited to Hierarchical clustering, GO, principal component analysis (PCA), pathway analyses and group analyses using T-test and Wilcoxon ran test. Hierarchical clustering was performed using the Euclidean distance metric and Average Linkage method. All of the samples used in this study were previously defined based on clinical diagnosis, receptor status and invasive potential as documented at Atcc.org and/or GEO. Both unsupervised and supervised analyses were performed in this study. Functional and pathway analyses were also assessed using NCBI, GATHER (http://changlab.uth.tmc.edu/gather/gather.py) and STRINGTM software (https://string-db.org/) in addition to mAdb.

## SUPPLEMENTARY MATERIALS FIGURES AND TABLES



## Abbreviations

BLIA: Basal-Like Immune-Activated
BLIS: Basal-Like Immune-Suppressed
C8orf46: Chromosome 8 Open Reading Frame 46
CCLE: Cancer Cell Line Encyclopedia
CD68: macrophage antigen
CD8: T cell antigen
CEL: cell intensity file
DHCR24: 24-dehydrocholesterol reductase
DMEM: Dulbecco's Modified Eagle Minimum essential media
ESR1: estrogen receptor 1
ETS1: ETS proto-oncogene 1, transcription factor
GATA3: GATA binding protein 3
GATHER: Gene Annotation Tool to Help Explain Relationships
GDS: GDS dataset
GEO: Gene expression Omnibus
GO: Gene ontology
GSE: GEO series
HER2: human epidermal growth factor receptor 2
HTLV: Human T-cell lymphotropic virus
IL-1ra: Interleukin 1receptor a
IL-1α: interleukin 1a
IL-1β: Interleukin 1B
IL32: interleukin 32
IL6: Interleukin 6
iL8: Interleukin 8
IM: immunomodulatory
LAR: luminal androgen receptor
M: mesenchymal
mAdb: microarray database
MES: Mesenchymal
MSL: mesenchymal stem–like
MYB: Avian Myeloblastosis Viral Oncogene Homolog
MYBL1: MYB proto-oncogene like 1
NCBI: National Center for Biotechnology Information
NFE4: Transcription factor NF-E4
NFKappa B: Nuclear Factor Of Kappa Light Polypeptide Gene Enhancer In B-Cells 3
PCA: principal component analysis
PCR: polymerase chain reaction
PD1: programmed death 1
PDL1: programmed death – ligand 1
PTX3: pentraxin 3
RAS/ERK: Rat sarcoma / Extracellular Signal-Regulated Kinase
ST6GALNAC5: ST6 N-acetylgalactosaminide alpha-2,6-sialyltransferase 5
STRING: Search Tool for the Retrieval of Interacting Genes/Proteins
TAM: tumor associated macrophages
TIL: tumor infiltrating lymphocytes
TMEM158: transmembrane protein 158
TNBC: Triple negative breast cancer
TNBC-25: Triple negative breast cancer Maire subclass of 25 patient samples
TNF: Tumor necrosis factor
TNFRSF: Tumor necrosis factor receptor superfamily
VEGF-STAT3: vascular-endothelial-growth-factor-signal-transducer-and-activator
