# Supplementary material for: Identification of candidate genes associated with triple negative breast cancer
Source: Genes Cancer. 2017 Jul;8(7-8):659–72. doi: 10.18632/genesandcancer.147 (PMC5620011; doi:10.18632/genesandcancer.147)
Supplement: Supplementary file 1 [file ganc-08-659-s001.pdf]

Identification of candidate genes associated with triple negative breast cancer

SUPPLEMENTARY MATERIALS

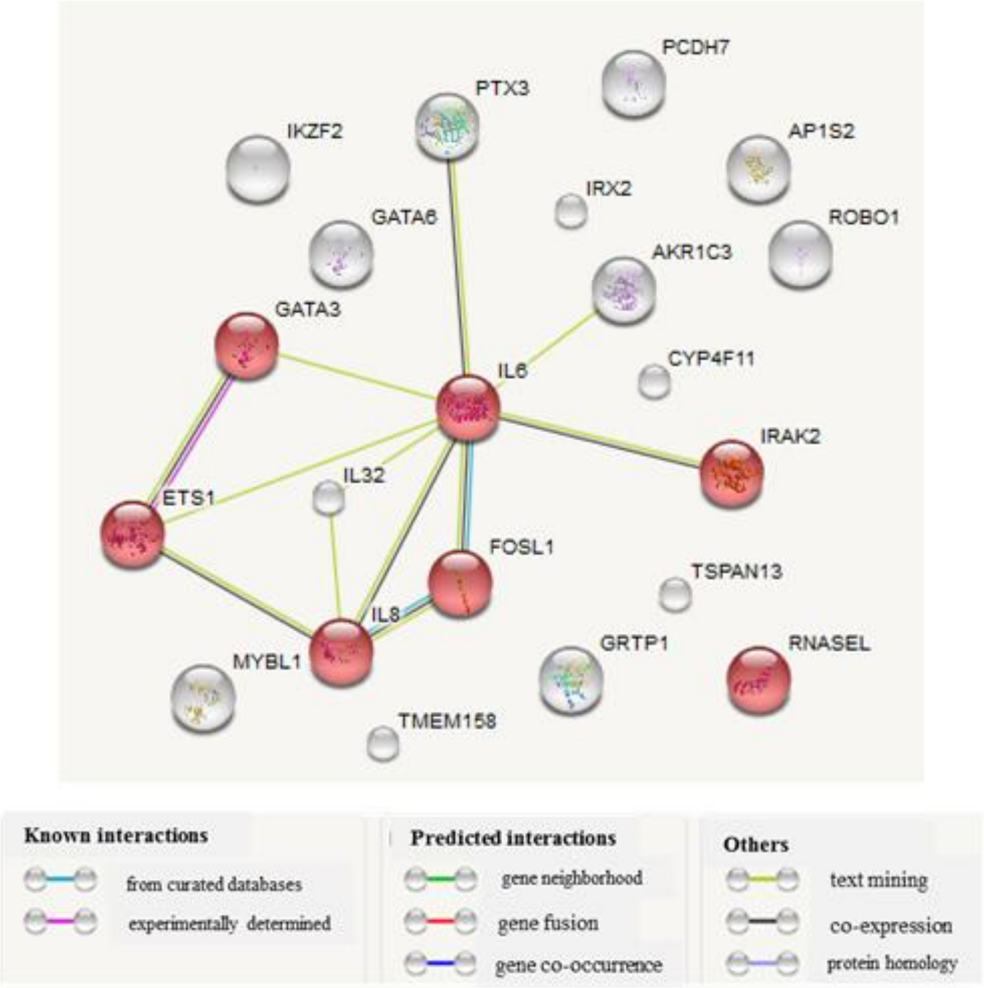

Supplementary Figure 1: String analysis of 21 gene list. Genes were identified by comparing subclasses of TNBC. a list of 21 genes were identified and examined using the STRING program (which takes into account all known protein information.

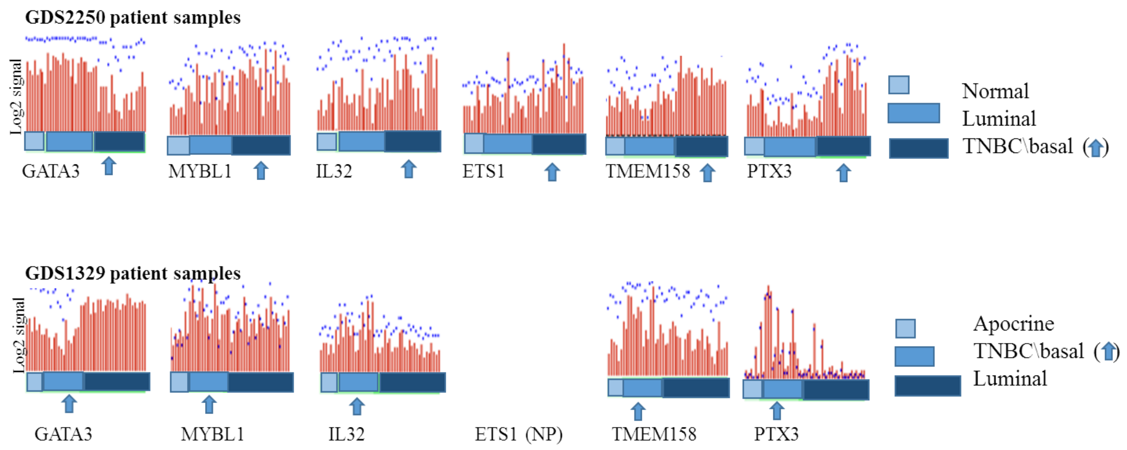

Supplementary Figure 2: GEO datasets GDS220 and GDS1329 showing the gene expression profile of the six candidate genes. ETS1 probe-set was not present (NP) in GDS1329 dataset.

Supplementary Table of 1: List of 21 genes identified as differentially expressed between the IL32-high compared to the IL32-low cell line samples. The upregulated genes (i.e., positive values) were expressed highest in the IL32-high cell lines. The downregulated genes (i.e., negative values) demonstrated higher levels in the IL32-low cell lines; pv= pvalue.

**Affymetrix probe-ID**	**Gene symbol**	**Fold difference**	**p value**	**Inflammatory**	**HTLV pathway pv 0.0031**
		**up-regulated**		**cytokine (pv-0.0001)**	
206157_at	PTX3	5.6	0.00039299	**yes**	no
202859_x_at	CXCL8	3.5	0.0062	**yes**	no
213338_at	TMEM158	3.3	0.017	no	no
205207_at	IL6	3.2	0.00365	**yes**	**yes**
213194_at	ROBO1	3.1	0.011	no	no
203828_s_at	IL32	2.9	0.0012657	**yes**	no
213906_at	MYBL1	2.8	0.026	no	**yes**
204420_at	FOSL1	2.7	0.028	**yes**	**yes**
1555355_a_at	ETS1	2.6	0.037	**yes**	**yes**
210002_at	GATA6	2.2	0.0062	no	no
230264_at	AP1S2	2.2	0.011	no	no
231779_at	IRAK2	2.1	0.011	**yes**	no

Affymetrix probe-ID	Gene symbol	down-regulated	p values	Inflammatory	HTLV
				cytokine	pathway
209160_at	AKR1C3	-3	0.011	no	no
228640_at	PCDH7	-2.9	0.02	no	no
231929_at	IKZF2	-2.8	0.0014	no	no
217979_at	TSPAN13	-2.4	0.019	no	no
209604_s_at	GATA3	-2.3	0.015	**yes**	no
206153_at	CYP4F11	-2.2	0.011	**yes**	no
229377_at	GRTP1	-2.2	0.04	no	no
228462_at	IRX2	-2.2	0.049	no	no
229285_at	RNASEL	-2.1	0.0059	**yes**	no

Supplementary Table 2- Description of the genes and corresponding PCR primer sequences

AFFYMETRIX	GENE	LEFT PRIMER	RIGHT PRIMER	AMPLICON
**PROBESET**	GAPDH	TCC CTG AGC TGA ACG GGA AG	GGA GGA GTG GGT GTC GCT GT	217bp
213906_at	MYBL1	AAG TCT GGG CTT ATT GGA CAT AA	TGC AAG TAT GGC TGC TAC ATG	202bp
209604_s_at	GATA3	GGT GTC TGT GTT CCA ACC AC	GTG GCC AGT GAA AGG AAA CA	295bp
1555355_a_at	ETS1	TCCAAAGAAGTTTCAAGGAACCA	TCTCTAAGCTACCTCAGTTCTGA	296bp
213338_at	TMEM158	TTC ATG GCA GAA AAT GAC CA	GGC CAC GTC ACT GGA TAG AT	286bp
203828_s_at	IL32	CCT GGA ACC ATC TCA TGA CC	AGC TGG AGG ACG ACT TCA AA	207bp
206157_at	PTX3	GTT GGG AAG GTC TGA AAA CTC A	ACA ATT GTC CCT CTG TTC AAC A	251bp
200862_at	DHCR24	CAG AGC CCT GAG TTT CTT GG	AGCCTGACAGGGAAGATTCA	203bp
1560527_at	NFE4	CTT TTA ACC CCA AGG ATG AAT G	ACA CAG TCA GAT CAG AAT CAG TGG	255bp
229430_at	C8ORF46	AAG AAG GGG CTA TCC CAG AG	AGC TAG GTG TGG TGG CAT GT	252bp
220979_s_at	ST6GALNAC5	TTG CCT TTG AAA CTG CAA CA	GGC CCA GAT TGC ACT AAA AA	262bp
